# The novel as therapy: Ministrations of voice in an age of risk

**DOI:** 10.5871/jba/003.035

**Published:** 2016-04-14

**Authors:** Patricia Waugh

**Affiliations:** Department of English Studies, Durham University, Durham, UK

**Keywords:** therapy, voice, the novel, ‘risk society’, recursive, mindreading, Bayesian brain, self-referentiality, fictionality, Witgenstein

## Abstract

Examining relations between ‘therapy culture’ and the ‘risk society’, this essay suggests that the novel developed to offer a powerful workout for the kinds of socio-cognitive capacities and gratifications required by the complex and ‘emergent’ cultures of modernity: recursive skills of mindreading and mental time-travelling, the negotiation of plural ontologies. Its development of a unique mode of ‘double voicing’ allowed readers to situate the interior life in a complex and dynamic relation to the social. Reading novels challenges the default, making ‘safe’, capacities of the probabilistic or Bayesian brain. In its self-referentiality and invention of the idea of fictionality, the novel provides an education into awareness of the limits of models and their dangerous fetishisation. The novel therefore answers Wittgenstein’s search for a discourse that might provide a therapy for errors in thinking, embedded deep in structural and analogical functions of language and especially those perceptual metaphors of vision that carry the epistemological beliefs that *looking in* is the route to self-transparency.

Artists are clinicians, not with respect to their own case, nor even with respect to a case in general; rather, they are clinicians of civilisation … It seems … that an evaluation of symptoms might be achieved only through a novel.^1^

Writing is controlled personality disorder […]. It’s controlled because in order to make it work you have to concentrate on the voices in your head *and* get them talking to each other.^2^

From the outset in formal philosophy, thinking has been thought of in terms of seeing … if one considers how easy it is for sight unlike the other senses to shut out the outside world and if one examines the early notion of the blind bard, whose stories are being listened to, one may wonder why hearing did not develop into the guiding metaphor for thinking.^3^

## The Small Personal Voice and the Complex Totality: Reading Novels

The novel *as* therapy rather than the novel *and* therapy or the novel *of* therapy. The title is intended as a challenge: to think through and then beyond some familiar assumptions about the nature of the novel and what might be understood by the term ‘therapy’ and to use the aspectival force of ‘as’ to generate some less obvious ways of seeing. The key concepts of the analysis will be those of complexity, fictionality, voice and recursivity. In the end though, everything rests on the peculiar *intimacy* of reading novels, their depiction of character as interiority; their capacity to absorb the reader through imaginary minds into a storyworld that takes on the feeling of the real even as it announces, in various ways, its fictionality. On the simplest definition of the novel, most would agree: novels are complex written narrative structures defined in contradistinction to other kinds of narrative by the peculiar and singular nature of their fictionality. But they are ‘told’, that is to say, mediated, not by a flesh and blood oral storyteller, but through ontologically and metaphorically slippery textual effects of voices. Novels are realised or ‘concretised’ in the imagination of their readers as storyworlds, in a controlled but often immersive experience that produces well-attested uncanny effects: the feeling that we know the places of fiction—Dickens’s London, Joyce’s Dublin, George Eliot’s Middlemarch—and their denizens—Esther Greenwood, Leopold Bloom, Dorothea Brooke—as well as or even better than the way we know the places of our own past or the personages of our present. Because the ontological status of the fictional world is determined by its verbal condition, novelistic worlds and their inhabitants are characteristically and tantalisingly ‘gappy’ or indeterminate. But so are minds. Reading novels, like the process of therapy, is the discovery through transference that the self is a dynamic process of intersubjectivity. But novels also offer us control: in the private space of reading and writing, characters appear exhilaratingly fresh and uncolonised, available therefore for the personal extrapolation of the reader in conversation with a real and yet imaginary author, a shadowy presence similarly reconstituted through the process of construal that is reading.

Not surprisingly, therefore, characters become companions, like childhood ‘imaginary friends’ who, mysteriously, never age but still grow and change with their readers, transition effortlessly back and forth across temporal and ontological divides. And in our immersion in them, whether as readers or writers, we enter a condition of absorption, a kind of controlled dissociative state. Close to the delusional and yet rarely crossing the line, all novel readers and writers know the experience of being immersed in a world that feels life-like in its unfolding of open horizons, each new perspective—whether the focalisation of a character or a new analogical trope that condenses a complex node of the experiential—seeming to add ‘depth’ to the whole. Yet all of this is experienced in the knowledge that nothing is risked, for we are safely held in the *telos* of the invented, offered space for the constant switching of outer and inner attention from the magical place opened up by the words, to the words themselves, and to the inner space of reflection, memory and planning, where we review our own wider experience. In reading fiction, through the entanglement with recognitions, identifications, challenges to our confirmation biases, we arrive at ourselves expanded through an encounter with the new and strange. We are made to review and therefore become more acquainted with our own expectations and wider assumptions, our own interpretative stance. And though this process, like any good *therapy*, can disturb, we are held in the singular mode of the fictional future anterior, looking forward in order to look back, experiencing that consolation of formal closure that offers retrospective meaning, and yet the feeling of existential openness as we move forward through the book: this is the import of Frank Kermode’s memorably phrased ‘sense of an ending’.^4^ Novels allow us to order our minds more completely by taking us closer to the edge of disorder.

The theme to be explored in the first half of this essay is that a powerful but overlooked aspect of the therapeutic effect of reading fiction lies in its transferential capacity to produce in us the effect, as in the ‘talking cure’, of a voice that mingles with our own inner speech, as if it listens to and throws back a more comprehensive and clarified version of what we take ourselves to be. In fiction, however, the transaction occurs internally, through the sounds as well as the pictures that we hear in our heads, so that attentive reading is a kind of mimicry of composition—as suggested in David Mitchell’s observation (quoted above) that writing novels is concentrating on the voices in your head and getting them to talk to each other. If composure, for writer and reader, is the outcome of composition, its achievement will almost certainly occur through disturbance or discomposure of our customary inner dialogue with ourselves. This is a process uniquely associated with the singularity of voice in the novel. Whereas the seeing self is separate from the perceived world, analysing its surroundings through the distancing eye, the listening self is immersed in that world through the sounds that reverberate through the ear and enter the body. Stephen Connor suggests that since we can hear many sounds at the same time, and cannot simply turn our ears away from a sound as we can our eyes from a sight, the ‘self defined in terms of hearing rather than sight is a self imagined not as a point, but as a membrane; not as a picture, but as a channel through which voices, noises and musics travel’.^5^ I will suggest that sound and voice are major agents for our feeling of immersion in fictional worlds, as important as ‘pictures’, but almost always over-looked in our tendency to think of consciousness as an ‘inner theatre’. Read attentively, most novels disturb the tendency to imagine an inner self that is unified and fixed, a shadowy homunculus, crouched in the dark, awaiting the searchlight of an inner eye that might illuminate and clarify its contours.^6^

In fiction, voice is a textual effect, often non-locatable in an embodied source. Often we are unaware who is speaking, for even the impersonal expressivity of style produces the feeling that behind the most objective description of a scene or landscape, or the apparently direct as well as indirect reporting of thought or talk, a ghostly enunciator hovers. This ‘voice’, like our own inner speech, might be heard only if we listen attentively: to tone, timbre, nuance, syntax, repetition. Mostly our attention is directed towards the world; mostly we barely notice our inner speech. In fiction, though, attention is drawn to voice as the necessary and complex medium of the story. Though novels often welcome us in pictorially, taking the inner eye over a threshold or along a path, often it is sound or voice that takes us in deeper to an imaginary world. At the opening of Faulkner’s *The Sound and the Fury* [1929], for example, we follow the uncomprehending Benjy Compson’s gaze ‘along the fence’, but what has brought Benjy to the perimeter of the golf course is sound, the repeated cry of ‘caddy’ that echoes in Benjy’s head, a name that makes emotionally present his long departed and disowned sister who, until her disgrace, had loved him with a kind of maternal fervour so lacking in the cold and self-absorbed Mrs Compson.^7^ Similarly, in the opening of Mrs Dalloway [1925] Clarissa’s transportation into the past of girlhood is effected through a train of thought, conveyed through free indirect discourse, where the word ‘hinge’—Rumpelmayer’s men are coming to take the doors off their hinges—brings an imaginary sound into Clarissa’s mind, a ‘squeak of the hinges, which she could hear now’, as the doors are opened and she ‘plunges’ into her young womanhood and the romantic complications at Bourton.^8^

If the voices of novels seem to speak to us intimately, the novel genre has long sought its defence in the humble terms of usefulness, entertainment or education, rather than in an elevated aesthetic of detached contemplation. Hardly surprising, therefore, that the novel has recently found itself appropriated for therapy. Prose is not Prozac. But one might be forgiven for thinking otherwise, given the boom in reading groups, six-figure-viewing TV shows such as the Oprah Book Club in the US, the Richard and Judy Book Club in the UK, or the considerable success and cultural impact of the Reader Organisation, launched in the UK in 1997, to promote shared reading as a therapeutic practice.^9^ Yet the novel’s widespread success was from the first almost entirely bound to the market and commercial fortune, subjecting it therefore to pressure to be morally edifying, sentimentally educational, socially connective: useful. If Scheherazade told stories to save her life, the first fictional character to be created without a prior model, Robinson Crusoe, is the ultimate icon of the survivor, talking to and writing himself into sanity and in so doing, creating, like his maker, a meaningful world, in a kind of *mise-en-abyme* effect that already echoes the multiple embedding of the oral tale in *The Thousand and One Nights*.

Crusoe is saved from the traumatic effects of his shipwreck and castaway condition by his ability to listen to his own thoughts, allow his inner voices to speak to each other, and in the comfort he takes in their externalisation as he talks to God and writes in his journal. He insists that his life, twenty-seven years in solitude, was ‘better than sociable, for when I began to regret the want of Conversation, I would ask myself whether … conversing with my own Thoughts, and, as I hope I may say, with even God himself … was not better than the utmost Enjoyment of humane society in the World’.^10^ Aside from the discovery of the footprint, his moment of greatest discomposure is when he is awakened from sleep by the voice of the parrot that has listened to his cries of despair and now mimics back his master’s voice, calling his name, ‘poor Robinson Crusoe’.^11^ Crusoe responds with terror; it is as if the inner voice of despair has broken free, to exert its independent agency as tormentor in the world outside his head. Joyce’s Bloom, some two hundred years later, walking through the Dublin cemetery to the funeral of Paddy Dignam and reflecting how ‘every Friday buries a Thursday’, finds the voice of the parrot now parodied in a ditty running through his head too: ‘O, Poor Robinson Crusoe, How could you possibly do so’ while his shade mysteriously reappears in the hallucinatory visions of Circe’s brothel fantasy.^12^ In Defoe’s novel, however, Crusoe mostly invokes the voice of the other within to control despair, addressing himself in the second person so as to challenge the loud expostulations of hopelessness with quiet admonishment, personified as the inner voice of reason: ‘Reason, as it were, expostulated with me t’other Way thus: Well, you are in a desolate Condition ’tis true, but pray remember, Where are the rest of you …Why were you singled out. Is it better to be here or there, and then I pointed to the sea?^13^ Does Crusoe actually or mentally point to the sea as he thinks about his drowned companions? Even in this earliest of novels, voice blurs the boundary between inner and outer, internalisation and externalisation: the pluralisation of inner voice allows Crusoe to listen to another voice that counters his downward spiral of thought; elsewhere voices allow him to externalise emotions he is unaware of so that he reflects, ‘I had a great deal of comfort within’; finally, he fully externalises his inner thoughts as a formalised dialogue in the double-entry book method of his journal, with its columns of ‘Debtor and Creditor’ that transform the religious discourses of sin into those of the new commercial world of credit.^14^ In creating the journal, therefore, Crusoe finds the means to resolve in himself, through the unique economy of writing, the conflicting voices conveying a world of miracle and revelation and the more austere rationality of Baconian method. The composition of the journal makes permanent the precariously achieved inner composure that is arrived at through ‘listening in’. In the early 18th century Crusoe has successfully discovered the benefits of ‘narrative therapy’.^15^

As the theory era gives way to the therapy culture and the preoccupation with narrative, affect and the body in academic criticism, the incorporation of the confessional novel into the more capacious category of ‘life writing’ has a well-established precedent therefore in the history of fiction. Scheherazade told stories to save her life but Robinson Crusoe, initially presented by Defoe as a true narrative, writes his way to survival. For the term ‘life-writing’ was coined by the Modernist scholar Shari Benstock^16^ to articulate the idea that if selves are processes generated through and entwined with endlessly looping and constitutive acts of naming, narration, assembling of voices, then selves, like novels, are not merely ‘narrative’—as in Bruner’s idea of self-narration as the defining act of the human subject—they are also, in some sense, fictional, made up. Not only are characters imaginary people, but people are imaginary characters.^17^ Under the umbrella of ‘life-writing’, memoirs, confessions, autobiographies and biographies have been gathered in alongside the crop of newer genres, the misery memoir, illness diary, autobiografiction, trauma testimony, the docu-novel and the new journalism, so that earlier distinctions between the factual veracity or apparent ‘truth’ of the memoir, and the evident fictionality or pseudo-referentiality of the novel—even without post-structuralist finessing—have grown blurred. But the so-called ‘narrative turn’ that has made writing synonymous with life and turned the talking cure into a writing one, has brought a further significant return.

After seventy years of New Critical textualism, modernist impersonality, affective and intentional fallacies, structuralist and post-structuralist banishment, critical interest has returned to the relationship between authors and the voices of their texts, and to readers and what they do with texts and the voices in them. Both might be regarded as quests for meaningfulness or imaginary conversation. The ‘hermeneutics of suspicion’ is lightening its scepticism. The ‘narrative turn’ has opened a pathway to the therapeutic. Even academic critics now acknowledge novels as a species of ‘life writing’ or writing that arises out of and feeds into the experiential; they have begun to feel that it might no longer be a betrayal of their professional dignity to write about how novels move real readers and shape their lives or to recognise the fact that authors are not just accidental precipitations out of intertextual collisions, but real beings who have toiled and left their imprints in the worlds made by words. The upshot is some loosening of the categorical distinction between the professional critic and the common reader: between reading novels for pleasure and intimate engagement and reading them as socio-political or philosophical critique, well-wrought urns. People read to be transported, to feel wonder, to understand themselves; the therapeutic turn has brought the engagement previously associated with the ‘common reader’ to the interpretative community of the academy, with its more formalist and historicist concerns.^18^ But with this affective and narrative turn come new worries: that we are all, literary intelligentsia and common reader alike, grown fuzzy and sentimental or—dare one say it—middlebrow—under the soft penumbra of the therapeutic. Academic criticism, however, always fears an inward or ‘subjective’ turn, away from social critique and ideological awareness to complicity with a confession-obsessed culture. So key issues are raised: are there ways of opening up the concept of therapy itself that might generate new means of framing and describing the nature and organisation of novels and facilitate new insights into their uses? If novels are therapeutic, how do they protect themselves or how might they be protected against reductionist practices of therapeutic appropriation or charges of inwardness, introversion or narcissism? How does the novel itself expand the possible modes and meanings of the therapeutic?

## The Novel, the Risk Society and the Rise of the ‘Therapy Culture’

What is a risk society? And what does it have to do with therapy and the novel *as* therapy? In World at Risk (2009), Ulrich Beck points out that the threat of illness, premature death, famine, plague and natural disaster was greater in the Middle Ages than in the 21st century. What has changed, however, is the semantics of risk. By the 17th century, ‘risk represents the perceptual and cognitive schema in accordance with which a society mobilises itself when it is confronted with the openness, uncertainties and obstructions of a self-created future and is no longer defined by religion, tradition or the superior power of nature’.^19^ The novel is born into this first modern risk society and is its literary expression. The novel was the first literary genre to create purely invented and singular ‘characters’ placed in complex and emergent storyworlds. The increasingly globalised world of the late 17th and early 18th century brought the first age of risk, credit and venture capitalism. For three hundred years, novels have examined how catastrophe and irreversible change arise mostly unpredictably out of the small and everyday turbulences of networks of human beings going about their daily business. Talk and thought, the modelling and negotiation of risk and probability, of threats and opportunities and of surprise and unintended consequences, is the very stuff of fiction.

The novel arose in an age of systems, situated both within and against system philosophies.^20^ It was contemporaneous too with the rise of the probability calculus, an age self-fashioning and reflexive, preoccupied with cultural capital and taste, the new economic precariousness of booms and busts. Requiring attention to social as well as economic performance, the negotiation of masks and a greater need for prediction of the behaviours of others, the age shared with the novel an awareness of the important role of affect in practical reason. For Lukacs, though never a cognitive map of a totality, the novel facilitated the modelling of the real beyond the lifeless statistics of the social sciences and the logico-empirical separation of facts and values.^21^ Its grasp of the complex and dynamic or dialectical nature of experience is reiterated and performed through the reading experience as it is mediated by the singularity of fictional voice. Novels might be seen as simulated models of worlds that are created through their own modelling activity, self-conscious testimonies to the power and the limitation of models in general and of their fetishistic tendencies: that we may be tempted to use them as vehicles for over-extrapolation when confronted with the new and unknown, taking selections as wholes and therefore blinding ourselves to the uncertainty principle always introduced by the model.^22^ Reading novels, therefore, might be thought of as a way of developing mental skills to meet the demands of this new risk society. Part of this involved the calculation of and judgement on probabilities, but also a recognition of the need to temper the purely logico-empirical account of knowing with a new awareness of the centrality of affect and embodiedness in negotiating a complex world. The novel has always been associated with the cultivation and strengthening of the ‘moral sentiments’ into what Michael Bell has described as a metaphysical principle, a bulwark against the Hobbesian account of competing interests in the war of all against all of the new market *homo economicus*.^23^ Novels provide training too in practical reasoning skills; in thinking beyond linear models of causality that reduce human and social processes to mechanical systems or discrete causes. That the real world is complex, messy and interconnected, self-interpreting and indeterminate; that it is always more than the sum of its parts, renders any single or linear model of causality grossly inadequate as the vehicle for explaining its emergent behaviours.^24^ Reading novels exercises the ability to grasp circular causalities, the way the world is experienced forwards and understood backwards, the way our confirmation biases trip us even as we think we are being most attentive, and the way our values and affective lives enter into what we deem to be our most impassive and objective judgements.

Even at the level of negotiating a storyline, novels offer a ‘workout’ for both linear and more complex kinds of thinking, requiring the exercise of hypothesis revision, inference, abduction, close observation, pattern recognition and the ‘looping’ effect of language and values.^25^ To follow a fictional plot hones recursive skills for mental ‘time travel’—projecting into the past and the future whilst relating the projection to a moving point in the present—in its dynamic and complex processes of prolepsis and analepsis. In reading novels, we are constantly required to realign the time of the story and that of the discourse in cognitive manoeuvres that involve numerous temporal embeddings. Similarly, recursivity as perspective taking, the necessity for and ability to model other minds, anticipate behaviours, reflect on motives is the essence of characterological intersubjectivity. Cognitive theorists such as Lisa Zunshine, Alan Palmer, David Herman and others have demonstrated how, by the 19th century, novelists such as Jane Austen had pioneered techniques for modelling six or seven embedded layers of meta-representation of other minds: that A thinks that B thinks that C thinks that A thinks that B thinks and on, demonstrating the capacity of the mind to track and monitor its intersubjective relations in minutely complex ways, but revealing also the vast possibilities for error, self-deception, self-delusion and subterfuge in the process.^26^

Indeed, invoking *Don Quixote* as the first modern novel, Beck notes that even the term ‘quixotic’ has come to mean the failure to apply to the modern world the mental calculations of probability that are required in its safe and successful negotiation. Don Quixote, trapped in inappropriate and rigid mental schema, is unable to respond to the new challenges of his time, the rise of a more complex and fluid and commercial world.^27^ His problem is that he mistakes model for real and though *Don Quixote* sets up the possibility of the novel as a genre, Cervantes’ work is really an investigation of the limitations of the heroic and chivalric codes for negotiating the modern world. Beck argues that ‘in the first modern novels this heroism of risk is narrated as an awakening into an unknown world involving ever more unpredictability’.^28^ But he suggests further that we now inhabit an age of *global* risk, an ecological network, whose cascading effects and recursive feedback effects outstrip in complexity the reach of even the most rigorous probabilistic calculation. Our terms of engagement change the nature of that process; even science now generates more uncertainties in its iatrogenic effects and unintended consequences than it produces effective models through which to grasp the dynamic systems of the new globalised era. Uncertainty, no longer overcome by knowledge, is more often its effect, in everything from the economy and consumer habits, to the spread of ideas and innovations, diseases and trends, climate and security systems. A world has opened up, according to Beck, without clear distinctions between knowing and unknowing; risk now ‘amalgamates knowing with non-knowing within the semantic horizon of probability’.^29^

Beck regards the novel as the genre which arose contemporaneously with the risk society. But he also connects the risk society to the rise of therapy culture. His key argument is that, since 1945, an increasingly globalised risk culture generates, fans and manipulates fears of mostly invisible and indefinable threats and with this a feeling of vulnerability, of being ‘at risk’, as a strategy for legitimising political and social control. The therapeutic becomes the vehicle for a political process which, since the Cold War, has sought to intensify belief in the separation of the public and the private in order that the escalating dysfunction that is the fallout of global risk society is experienced and represented as privatised and interior. Suitably privatised, malaise is then medicalised as part of the management of affective life. Frank Furedi takes Beck’s argument as the starting point for his book, *Therapy Culture: Cultivating Vulnerability in an Uncertain Age* (2004), where he argues that the risk society is the major agent in the rise of ‘therapy culture’. Furedi’s concept of a ‘therapy culture’ draws on Philip Rieff’s earlier account of the ‘triumph’ of the therapeutic.^30^ Rieff’s analysis, of the therapeutic as the epistemological and ethical centre of instrumental rationality, grew out of and influenced the work of Herbert Marcuse, Christopher Lasch and Thomas Szasz in the 1960s; since 1980, it has been taken up by commentators as politically various as Alistair MacIntyre, Richard Sennett and Fredric Jameson. All, however, share a view of the ‘therapeutic’ as the velvet glove of an increasingly managed and bureaucratic society. This is the key theme that sustains Alistair MacIntyre’s seminal study of moral theory, *After Virtue*, published in 1981.^31^ Furedi focuses therefore on the medicalised management of everyday life and, in particular, on the generation of ever more diagnostic categories to explain the human experience of distress or malaise. Indeed, the year before the publication of MacIntyre’s book, the American Association of Psychiatry published the third edition of its diagnostic handbook (*DSM*, III, 1980) which included a new syndrome, Post Traumatic Stress Disorder, or PTSD, soon to become a buzzword of the post-1980s generation, along with its constituent terms, ‘trauma’ and ‘stress’. The term ‘stressed out’ entered colloquial language in 1983 at the heart of what would be termed the ‘postmodern condition’, with its avowed sense of the ‘unmappability’ and disorienting complexity of an increasingly globalised and uncertain world. The deregulation of financial services and the caricature of the burnt-out city executive were key themes in the fiction of the decade: in the work of Don DeLillo, Brett Easton Ellis and Martin Amis.^32^ The novel, the risk culture and the therapeutic, seem to have grown up together, evolving hand in hand. When one considers that the fifth edition of the *DSM* now lists 500 syndromes, then it is hardly surprising, as is evident glancing over the *Pocket Guide to Therapy* (2012) with its praise for the greater ‘diversity of therapy’ and the professionalisation of its models, that there are now dozens of therapies from which to choose: from cognitive behaviour therapy, cognitive analytic therapy, psychodynamic therapy, systemic therapies, narrative therapy, person-centred therapy, couples therapy, family therapy, mindfulness, solution-focused brief therapy, dialectical behaviour therapy—and many, many, more.^33^

In this context, Furedi’s argument that the age of the ‘talking cure’ has also been the age of a professionalised therapeutic *cultivation* of vulnerability, rather than the provision of a means for its overcoming, has some force. Furedi describes a situation where a now all-pervasive therapy culture perpetuates and disseminates itself by inventing ever more syndromes in a general pathologisation of experience that translates most of the challenges of living in an uncertain world into the vocabularies of psychological medicine: ‘distress is not something to be lived but a void that needs treatment’.^34^ Like Beck, too, he reverses the normally assumed relations of cause and effect, viewing the concept of ‘vulnerability’ at the heart of the therapeutic as an invention scaffolded by the promulgation and dissemination of the concept of ‘risk society’. Instead of *taking risks*, understood as using our resourcefulness to meet the challenges that life throws up, we see ourselves increasingly *at risk*, vulnerable, in need of professional intervention. Everyone complains endlessly of being ‘stressed’ and every difficult or challenging experience is read as ‘traumatic’. As the world grows more complex and unpredictable and as the risk discourse reinforces a sense of things spinning ever beyond our control, individuals are recast as powerless victims-in-the-making: ‘today’s tendency to interpret events through the prism of trauma serves to cultivate a profound sense of fatalism in the public imagination’.^35^ And as the world becomes ever more complex and beyond the predictions of probabilistic reasoning, the individual is encouraged to see his or her problems as internal and fixed: the consequence of irrational thinking or uncontrolled affect. The recourse to professionalised and ‘expert’ intervention is more and more sought. For Furedi, the possibility of cultivating resilience, self-composure and an activist stance through shared and meaningful exchange within a community, therefore slips out of view, an abandoned fantasy of the ‘organic community’.

Novelists themselves have expressed concern about their often unwilling recruitment to this new therapy culture. Caryl Philips, for example, worries that writers are in danger of becoming the new face of ‘care in the community’, a fear brilliantly and surreally played out in Kazuo Ishiguro’s provocative and inventive novel of 1995, *The Unconsoled*, with its examination of the fortunes of the contemporary artist, expected to be part of a cosmopolitan world of ‘caring professionals’, to be an international ethicist who must exercise a kind of impossible telescopic philanthropy as he is compelled to respond to local demands in a globalised world.^36^ Commercial forces control his schedule and global demands consume his rehearsal and performance time; he has become a stranger to his family and loved ones, but mostly to himself. But Ishiguro, like many writers before him, has also averred the therapeutic value of writing fiction as consolation for the wound incurred in the passage from a naive narcissism to a more mature reality principle, the recognition that ‘the world isn’t quite the way you wanted it but you can somehow reorder it or try to come to terms with it by actually creating your own world’.^37^ In David Lodge’s comically Kierkegaardian novel, *Therapy* (1995), his mid-life crisis protagonist, Tubby Passmore, tries out a smorgasbord of therapeutic consultations and cures and various erotic distractions until he recovers his soul, if not the ‘internal derangement of his knee’, through the decision to keep a journal. Like his author, and like the first fictional character of the novel in English, Tubby, we assume, has saved himself through writing.^38^

Of recent conversions to the therapeutic, Jonathan Franzen’s has received most publicity. In an infamous essay published in *Harper’s Magazine* in 1996, entitled ‘Why Bother?’, he insisted that he couldn’t ‘stomach any kind of notion that serious fiction is *good* for us’, explaining that, ‘it’s hard to consider literature as medicine, in any case, when reading it serves mainly to deepen your depressing estrangement from the mainstream; sooner or later the therapeutically minded reader will end up fingering reading itself as the sickness’.^39^ But Franzen is now one of the converted, advocating the novel as a kind of panacea for a society of the ‘lonely crowd’, defending fiction as one of the few freely available antidotes to the kind of existential loneliness that is at the heart of so much contemporary distress and suffering. Reading fiction offers the promise of a communion of minds; he now insists that he writes, ‘to find an adequate vehicle for the most difficult stuff at the core of me, in the hope that that might resonate in the reader who otherwise has been feeling alone with those feelings’.^40^ Franzen uses the vocabularies of contemporary therapy but his defence of the novel is as old as the genre. From the early 18th century, the legitimacy of the new genre rested on its capacity to defend itself from charges that in immersing its readers in imaginary though credible worlds, the novel might ‘disorder’ minds and contribute to delusional states.^41^ This fear continued until novelists became comfortable with the ontology of *fictionality*, no longer feeling the need intentionally to blur the boundary between the imaginary and the real, to dress up their stories as ‘life’ writing in the form of purportedly ‘true’ memoirs, confessions, journals. Unease about the deceptivenss of the new form, its probabilism and credibility, lay in fears that the novel was being designed intentionally to bamboozle readers into taking the imaginary for the real. The counter-argument to this rested on seeing novels as a moral force, able to organise and refine the moral sentiments and contribute to the development of the capacity for fellow feeling or empathy.

Franzen, of course, uses fictionality in his own novels as an ironic tool with which to challenge easy complacency in the sphere of the empathetic, the lurking narcissism that can lie in an over-ready *caritas* with its potentially self-gratifying display of altruism. But this kind of affective irony is not a singular native of postmodernism, for it was actually an invention of the 18th century. Here we return to the importance of voice in any evaluation of the relation of the novel to the therapeutic. In Sterne’s *A Sentimental Journey Through France and Italy* [1768], its author plays with the ambiguities of language, so that the vehicle that transports its sentimental traveller is also that of the book we are holding in our hands. Of the various staged moments of sentiment, the most ironic is the scene where Yorick sees the bird in the cage, languishing in its confinement, so he wills himself to stand before it and imagine that it represents ‘the millions of my fellow creatures born to no inheritance but slavery’. But having indulged the moment of fellow feeling, Yorick runs off, overwhelmed, leaving the poor bird to be sold into a further and more abject condition of slavery. Sterne is evidently deploying irony to reveal the limits of the sentimental and its precarious closeness to the self-regarding emotionalism of narcissism. If we examine the passage more closely, however, what is evident is the emphasis on the way that narcissistic sentimentality is linked specifically and quite emphatically to *visual* perception, to the act of looking and seeing; we are told how ‘affecting’ Yorick finds ‘the picture’ of the bird, how he looks closer through the cage door ‘to take his picture and how he there ‘beheld his body’. It is only when in imagination, in his inner *ear*, that he ‘heard his chains upon his legs’ that he was then able to see ‘the iron enter his soul—I burst into tears—I could not sustain the picture of confinement which my fancy had drawn’.^42^

Though the sentimental novel is usually viewed as ironising the excessive sentimentalism of benevolent philosophy, the most influential treatise on empathy, Adam Smith’s account of the moral sentiments, shares with Defoe, Sterne and other novelists such as Richardson the emphasis on inner hearing rather than outward seeing as the source of the truly empathetic; for it is in the inner speech of thought that the meaning of suffering might be processed, as in the operation of the still small voice of conscience as opposed to the immediate sensorimotor ‘vibration’ triggered by the visual spectacle. Attunement to the inner voice as a kind of internalised hearing of and sustained reflection on the voice of the other is foregrounded. Listening in is here the basis for understanding the mind of the other rather than any sympathetic vibration or mirroring in the nervous system. That glimpse of real suffering, however, is too much for this particular sentimental tourist to bear.

But the mediation of the novel through the unique development of ‘voice’ enables an alternative therapeutic value to Furedi’s long arm of the managerial and the instrumental that he sees as professionalised therapy. In reading a novel, it is the small personal voice that connects the individual self to the generation of a complex world with its multiple points of entry, focalisations, perspectives and meta-representations (the modelling of one mind inside another). The association with ‘therapy’ in the original sense of *therapia*—understood as to ‘minister to’ or provide remedy for—is implicit in the common analogy of both reading and writing novels with the fundamental modern therapeutic situation of the ‘talking cure’. Like that of the therapist, the ‘voice’ of the novelist heard through the voices of characters produces an uncanny sense of felt presence when it enters, disturbingly or consolingly, into the internal conversation that all of us have with ourselves, as we reframe and filter our experience. To read a novel is a formalisation of this fundamental way of processing our lives that requires our full and sensitive attention but neither therapist nor, indeed, critic. The view taken here is that the novel might be said to amplify and strengthen the self’s capacity for negotiating and living in, as well as examining, the terms of what Beck has called the ‘risk society’.

My argument is that the key vehicle for this process is the unique use and effect of voice in the writing and reading of novels. As Miguel de Unamuno noted in 1913, ‘to think is to talk with oneself, and each of us talks to himself because we have had to talk to one another … Thought is interior language and internal language originates in external language’.^43^ In novels, we talk to ourselves mediated through the voices of the other but continuously jolted back into an awareness of our own inner voices as we negotiate textual hermeneutics through the prism of our own personal memories and readerly experience. As Denise Riley suggests, ‘If I swing my attention onto my inner speech, I’m aware of it *sounding* in a very thin version of my own voice’.^44^ In reading we hear more insistently and become aware of thought as a play of voice, but we also experience a kind of auditory decentring and realignment as the ‘voice’ of others enters our own; we become aware too of how what is ‘me’ is always already constituted out of the voices of the other and that the monitoring of what is me is thus a complex and fragile process, a kind of constant performance of auto-ventriloquism, always threatening to break down with the arrival of new or previously unheard voices. But that is how inner speech functions as thinking outside fiction, though we mostly forget that the ‘voices’ we hear as our own in the flow of inner dialogue with ourselves are those decompressed internalisations of the dialogue we take in from birth, from the world outside.^45^ That is why novels remind us continuously, as Bakhtin argues, that language is ‘not an abstract system of normative forms but rather a concrete heteroglot conception of the world’.^46^ Voice in fiction is a revelation that the self outside fiction is not simply a self-interpreting narrative but, even before this process of reflection and ordering, already and fundamentally a heteroglot of voices. If we pay attention to the complexity of voice in fiction, we realise that, in being most psychologically attuned to interiority, the novel is also most attuned, at the same time, to social context.

Recent research on reading fiction suggests that internal attention is directed through voice: we tend to hear our own inner voice when the language of the text is complex or challenging and demands attention or resonates with other motifs and passages as in the construction of a sense of spatial form.^47^ In dialogue, we hear the imagined voices of characters or an absence of auditory voicing as in more discursive or information giving passages of writing. Reading is again a kind of formalisation of thinking: the internalisation of dialogue that produces our inner speech, much of it condensed into the form of a kind of silent telegraphese sometimes referred to as ‘mentalese’, is re-elaborated as we consciously turn our attention to difficult tasks, or to reflection on our ongoing flow of perceptual experience. Here we hear our own inner voice as an internal dialogue or inner speech. So in reading, as we struggle for meaning through the opacity of the medium—its resistance to ready-made absorption and its obtruding of itself, the inner voice identified as our own, struggling to create meaningfulness, plays in and out, hearing itself in meta-interpretative activity, hearing the other in dialogue and interior monologue and the sounds of the imaginary world, as the medium seems to become transparent. Understood in such terms, we can see that the capacity of the novel to present ‘interiority’ is also its capacity to present the way in which the voices of the world shape and determine what we hear inside our heads. So the therapeutic qualities of the novel are not dependent on a narcissistically focused introspection or an ‘inward turn’ that takes no account of the pressures and contexts of the world. As Stephen Toulmin has argued, this is to confuse interiority with inwardness: ‘our mental life goes on in the interior; it just happens to be the business of neural networks in the cortex rather than of some metaphysical soul’ but, he continues, ‘our lives become inward because we make them so’.^48^ In Beckett’s novels, for example, the turn inwards is a consequence of a world that no longer offers scaffolding for a self; the internal dialogue has so far replaced all sense of anything outside that the voice speaking or the self writing no longer knows whether it speaks or is spoken, writes or is written:

Where would I go, if I could go, who would I be, if I could be, what would I say if I had a voice, who says this, saying it’s me? Answer simply, someone answer simply. It’s the same old stranger as ever, for whom alone accusative I exist, in the pit of my inexistence, of his, of ours, there’s a simple answer […] I’m not in his head, nowhere in his old body, and yet I’m there, for him I’m there, with him, hence all the confusion. That should have been enough for him, to have found me absent, but it’s not, he wants me there, with a form and a world, like him, in spite of him, me who am everything, like him who is nothing. And when he feels me void of existence it’s of his he would have me void, and vice versa, mad, mad, he’s mad.^49^

All of Beckett’s fiction plays out the consequences of turning inward when the world refuses to listen and, though the idea of Beckett as a Cartesian parodist is less prominent in recent criticism, there is no doubt that Beckett appears intentionally to displace the Cartesian metaphor of knowledge as seeing and seeing in, of transparency and clear and distinct ideas, with one of listening and listening in to a deeper unknowing.^50^ Talking—either inside the head or outwith—is never regarded as a cure by Beckett or his characters. Writing is here a necessary but barely remedial act in the face of the enormity of the world’s suffering.

## Doing Stress in Different Voices: The Novel and the Talking Cure

Not all novelists have responded quite so bleakly to the idea of a ‘talking cure’: the idea that emerged in the English speaking world after Freud’s successful American visit of 1909. In this section, I will briefly examine two writers—Wells and Woolf—whose work, in responding to the first stirrings of ‘therapy culture’, offers insights into the therapeutic possibilities of the novel at the dawn of the new and complex ‘risk’ society that began in the early 20th century. In the opening chapter of H.G. Wells’s *The Secret Places of the Heart* (1922), Sir Richmond Hardy, suffering from what would now most likely be diagnosed as minor depression or ‘stress’, consults his Harley Street physician, Dr Martineau, in search of a ‘pick-me-up, a stimulating harmless drug of some sort’. He is looking, he says, for something that will ‘pull me together’, get him ‘up to scratch’ again: ‘I’ve lost my unity. I’m not a man but a mob. I’ve got to recover my vigour. At any cost.’^51^ The good doctor, however, declining the request for a ‘tonic’, diagnoses a disorder of *thinking*; his patient is living too much in his head, he advises, ‘the current of your thoughts fermenting’, no longer able to reach beyond the skull and infer the direction of the world. Martineau’s recommendation is homeopathic: ‘why go out of the mental sphere for a treatment? Talk and thought; these are your remedies.’^52^ But he also reassures Hardy that his is not a singular aberration; his maladaptive ruminations are manifestations of a collective condition infecting his entire generation. Before 1910, he insists, the experience of the world was of a ‘sheltering and friendly greenhouse in which we grew. We fitted our minds to that … And here we are with the greenhouse falling in upon us lump by lump, smash and clatter, the wild winds of heaven tearing in through the gaps.’^53^

In this new world, malaise is the norm, morbidity generalised, disaster felt to be imminent: ‘this sense of a coming smash is epidemic … It’s at the back of all sorts of mental trouble. It’s a new state of mind. Before the war it was abnormal—a phase of neurasthenia. Now it is almost the natural state … a loss of confidence in the general background of life. So that we seem to float above abysses … A new, raw and dreadful sense of responsibility for the universe. Accompanied by a realization that the job is overwhelmingly too big for us.’^54^ In 1922, such professional discussions of shock, stress and trauma were extended beyond the Harley Street consulting room to enter parliament, with the presentation of the *Report of the War Office Committee of Enquiry into ‘Shell-shock’*, given significant press coverage in the Autumn of that year.^55^ Virginia Woolf’s *Mrs Dalloway* (1925), though not published until three years later is set in 1923, a few months after the report. The character of the war veteran Septimus Smith is evoked through the inner voices of his thought in contrapuntal and distributed entanglement with the voices of a war damaged London society struggling to recover its equilibrium. Like Joyce’s *Ulysses*, this is a novel that axiomatically foregrounds the centrality and therapeutic value of voice in fiction whilst it offers the first extended analysis of the aetiology of trauma, personal and public, in its theme. Four years after Armistice, Septimus haunts the edges of the metropolis whose familiar sounds, the ‘swing, tramp and trudge … the bellow and the uproar … the triumph and the jingle, once so exhilarating, are now a dissonant “clanging”, and whose customary rhythms, “the leaden circles” dissolving into air’, have turned stochastic.^56^ Like Defoe’s Crusoe, or Coleridge’s mariner or Conrad’s Marlow, Septimus is another revenant, called forth to tell his tale: ‘“To whom?” he asked out loud. To the Prime Minister, the voices which rustled above his head replied … painfully drawing out these profound truths which needed, so deep were they, so difficult, an immense effort to speak out.’^57^ Like Wells’s wise doctor, Septimus too intuits that the path to recovery might lie in ‘talk’ and ‘thought’: ‘communication is health, communication is happiness’, he thinks.^58^ But as he talks with his voices, others overhear, but rarely listen; wife and doctors alike dismiss his ‘message’.

The report on shell-shock, published four years after Armistice, might be regarded as the first official license for the ‘talking cure’. For it was the first official recognition that recursivity, self-interpretation, self-talk, is as much part of the complex aetiology of trauma as the simple causality of a physiological response to discrete physical or mental shocks. Trauma eludes simple causal explanation and requires the grasp of dynamic complexity or emergence; for its devastation lies in the seismic ripples of aftershock that serve retrospectively to amplify and multiply the effects of the original shock which may then settle into a circle of intensified interpretation that takes on characteristics of the delusional. Even relatively small disturbances may begin a process eventually running on its own internal momentum that leads to shattering of the self. Trauma theorists such as Kathy Karuth and Judith Herman, drawing on the pioneering work on dissociation of the psychiatrist Bessel van der Kolk, have argued that for the trauma victim, the repetition-compulsion so evident in the persistence of Septimus’s voices in calling him back to the scene of war, arises not simply out of the shock of the threat of death, but more as a response to the missing of the experience, the sense that the trauma response of numbing has not allowed the victim to know and therefore know how to incorporate the event into a meaningful narrative.^59^ The defence of dissociation, or numbing, like the terrified crouching animal caught in the headlight, protects against annihilation by the overwhelming force of the original offence. What has been blanked, however, comes back to haunt, uncontrolled, like the ‘tip of the tongue’ experience, where absence is felt as presence. Woolf is the first to experiment with fictional multi-focalisation, formal and recursive patterning and repetition, networks of echolalic sound and metaphor, in order to immerse her readers in a formal enactment of traumatic experience as a complex and emergent process. Furthermore, she achieves much of this through experimentation with voice. All the organising tropes of the novel are auditory: shouts, rustlings, reverberations, echoes, amplification, leaden circles dissolving into air. Woolf reminds us that sound—unlike sight which can preserve detachment, the capacity to shut down at will—infiltrates the body, just as the voices of memory blur past and present. Septimus loses all sense of the matter that is him and that is other.

For Woolf’s novel too shows how anomalous beliefs take on the force of incorrigible truths as they are employed to salvage some meaning, model new patterns of coherence, for a world whose faiths have shattered; she uses metaphors of reading and writing—the sky-writing plane, Septimus’s ‘message’, his inability to ‘read’ the world—and engages her readers too in reflection on their own hermeneutic activity, the process of hypothesis, revision, inference and tropic formulation whereby we assign meaning in reading both novels and worlds.^60^ For this complex process of making meaning, even as it flips into the mode of the delusory, reflects the workings of singular consciousnesses always infiltrated and shaped out of the internalised voices of custom, family and culture. This process of enculturation and individuation, defying capture through top down description, ‘naming’ or analytic reduction, is encountered in the reading of fiction as an experiential process. Never before had the processes and effects of what, in today’s parlance, would be diagnosed as post traumatic stress, been so authentically captured as an unfolding experience that also carries a self-referential awareness of its own complexity. Certainly, the concept of stress as a failure of adaptation to or a sense of being overwhelmed by the new and accelerated forces of modernity was already being described in neurobiology and medicine in the early twenties.^61^ And Hans Selye’s later notion of a ‘general adaptation syndrome’ came to underpin the bio-medical study of stress by the mid-20th century, drawing on and informed by cybernetic systems theories.^62^ But developing the novel’s capacity to model complexity, Woolf intuits, as early as 1925, and well before medical recognition, the emergence of traumatic experience through a complex dynamic process that she is able to model by developing the formal resources of the literary novel. Reaching beyond the homeostatic and the bio-regulatory, Woolf recognised the way in which the complex internal feedback processes in the aetiology of traumatic illness are interconnected with numerous social and cultural systems that amplify and intensify its effects. She insists to herself: ‘I want to criticise the social system, and to show it at work, at its most intense.’^63^ Medical psychiatry in the 21st century is only just beginning to appreciate the emergence of traumatic illness in similarly formal and dynamic complex terms.

Though Woolf and Wells consider the relationship between global conflict, economic instability and the more indefinable effects of ‘mood’ on individual well-being, neither presents the professionally therapeutic models of their time as appropriate responses to the suffering mind. Woolf famously eschewed therapy.^64^ She regarded reading and writing, however, as therapeutic; indeed, the term bibliotherapy first entered the English language in 1916, in an article published in *Atlantic Monthly* entitled ‘The Literary Clinic’. But Woolf is also the first writer to try to understand why the alchemy or magic transformation of writing might be as or more effective than the transferences and counter-transferences of analytic therapy. Words, she suggests, germinate something in the self that can feel as real and material as flesh itself, producing an uncanny sense of intimacy in the evocation of place, character and mood. Woolf writes fascinatingly of the thin line between fictionality and delusion, thoughts that are shaped into the controlled and written expression of voice and those that seem to explode out of the head or race ahead of one, taking on a life of their own.^65^ She writes of the way words can seem to have magical powers: the ‘feeling of transparency in words when they cease to be words and become so intensified that one seems to experience them; to foretell them as if they developed what one is already feeling’.^66^ That is why the written word, more than ‘talk’ and ‘thought’, has the power to ‘compose’ the affective life, to soothe and order the mind and make the fleetingness of thought into a permanent whole. In her 1927 essay, ‘Life and the Novelist’, she dismisses writing that is ‘soft and shapeless with words … upon real lips’ as giving no relief from the ‘swarm and confusion of life’. The art of writing must have ‘backbone’, ‘something compelling words to shape’; indeed a novel might be thought of as ‘Chinese coat able to stand by itself’. She writes how, in the discovery of that ‘design’ which compels words into shape through writing rather than thinking and talking, ‘there emerges from the mist something stark, something formidable and enduring, the bone and substance upon which our rush of indiscriminating emotion was founded’.^67^ Bodily synecdoche evokes her magical relation to the written, words chiselled out of thoughts that become flesh.

Few writers though have reflected so intensively on the complex possibilities of ‘voice’ in fiction that might allow the conversation with oneself that is inner speech to be refined into an inner dramaturgy.^68^ Indeed, Woolf often describes, through free indirect discourse or thought reporting, the effect of a character suddenly turning attention upon her own thought heard as inner speech: as Mrs Ramsay settles herself, the children retired to bed, in the first section of *To the Lighthouse*, for example,

often she found herself sitting and looking, sitting and looking, with her work in her hands until she became the thing she looked at—that light, for example. And it would lift up on it some little phrase or other which had been lying in her mind like that—‘Children don’t forget, children don’t forget’—which she would repeat and begin adding to it. It will end, it will end, she said. It will come, it will come, when suddenly she added, We are in the hands of the Lord. | But instantly she was annoyed with herself for saying that. Who had said it? not she; she had been trapped into saying something she did not mean … What brought her to say that …?’^69^

The passage again shows how fiction can blur inner and outer: in the phrase ‘she had been trapped into saying something she did not mean’ and in ‘Who had said it?’, the text is presumably referring to Mrs Ramsay’s inner speech, the train of her thought as the unwanted voice of patriarchal religiosity pops up unexpectedly. Or is it that we are listening to Mrs Ramsay speaking out her thoughts, as in Defoe’s *Robinson Crusoe*?

Though Woolf admired Defoe, her early experiments with voice and inner speech appear to have been most influenced by her early enthusiasm for Dostoevsky’s ability to capture ‘those most swift and complicated states of mind, of rethinking the whole train of thought in all its speed … to follow not only the vivid streak of achieved thought, but to suggest the dim and populous underworld of the mind’s consciousness where desires and impulses are moving blindly beneath the sod’.^70^ It was also after reading Dostoevsky that one of the most influential modern theorists of the novel, Mikhail Bakhtin, developed his own theory of ‘double voicing’, where, he argues, the author ‘uses another voice by inserting a new semantic intentionality into a discourse which retains an intention of its own’.^71^ He suggests that what is unique about voice in fiction is that one or more voices occupy another creating a ‘double voice’; fictional writing turns into creative capital what R.D. Laing would later call the ‘phantom concreteness’ of thought-fusion. A voice is always inhabited by the ghostly trace of another.^72^ Woolf, herself a voice hearer, acutely aware of the proximity of sanity and insanity, refers to the ‘phantom’ of thought in her essay on Montaigne, but she knew more than most how the voice that sets up its tenancy within the voice that is recognised as one’s own, as Bakhtin puts it, ‘acts upon, influences, and in one way or another determines the author’s discourse’.^73^ Many writers have spoken of how voices seem to arrive with agency, asking to be shaped into characters, but for Woolf, the internal wrestle for self-possession, the battle with the voices that so often seemed to run away with her, relied on a capacity for fiction-making that was, undoubtedly and literally in her case, sometimes an act of survival. Woolf recognised just how much the negative capability at the heart of self-recognition, the capacity of the writer to become another, is both a blessing and a curse.

## A Wittgensteinian Remedy: The Novel as Therapy for the Therapy Culture

The novel might be considered the most effective instrument the modern world has evolved for exercising all aspects of the ‘social brain’, or practical reason, the skills required for negotiating the modern world of uncertainty and complexity. Novels reveal the stress of anticipating and dealing with the unexpected, the trauma of its arrival in forms that often only retrospectively fit a pattern. Cognitive scientists now believe that the brain is a Bayesian mechanism, equipped for probabilistic reasoning. Though located inside the head, it reaches the world through the affective and sensory feedback of perceptual error when the world fails to fit the schema projected onto it; from brain to emergent properties of mind, this process allows the modification of existing schema in order to produce a more effective fit as the world changes.^74^ The brain is continuously monitoring the gap between the schematic hypotheses put to the world and the data that the world feeds back. Novels model some aspects of the world beyond, in their own storyworlds—realistically, fantastically, analogically, allegorically—and require readers to negotiate the complexity of the storyworld through their own error correction in reading, at the same time requiring that they acquire the appropriate schema for assessing and negotiating this particular storyworld. Here, negotiation of the ‘gap’ is also that between the imaginary world of the novel and the reader’s own historical context. Carefully read, novels educate their readers in how to read them by offering a workout for Bayesian skills first recognised and then developed in the 18th century, the first age of risk. But novels also offer further education into awareness of the shortcomings and limitations of probability thinking. The Bayesian brain might be understood as configured to reduce uncertainty by providing a default ‘safe’ system, reliant on habit and routine, the broadly predictable learnt from previous experience and involving the use of analogical reasoning, inference, extrapolation, in order, above all, to avoid surprise as evolutionary costly. But the Bayesian brain is a poor instrument for coping with shock, trauma and surprise, that is, the radically unexpected. The kinds of extreme and sudden change, associated with the critical transitions of ‘tipping points’, arising in complex and interconnected and emergent systems reveal the limitations of the Bayesian; but surprise and the unexpected is the lifeblood of novels. The cascading effects of small actions, the circular and backward causalities that amplify and intensify through a complex system, are all familiar to novel readers; as the American novelist William Gass observes: ‘it’s the daily diet—angers, fears, humiliations—Dr. Johnson’s tea, Balzac’s coffee, Freud’s cigars—which lead the liver to overlabor, stomach to puncture, heart to fail, the quiet worker to go berserk and ghetto to erupt, though it’s only the seizure, stroke, or strike which reaches the papers’.^75^

Novels keep the Bayesian mechanism sharp through the challenges, in reading, to confirmation bias, faulty inference, inattentive focus; but they also reveal the limitations of all models and suggest how, when prediction and knowledge systems fail, in an increasingly uncertain world, emotional and practical skills of resilience will have to compensate for the effects of unknowing. One thematic preoccupation of the novel has always been with the propensity of the human mind to protect itself from the threat of overwhelming trauma by hiding from itself the signs that small changes are building towards potentially more serious or even catastrophic proportions. Novels have always been fascinated by the delusional, the self-deceptive and the failure to read the signs of oncoming disaster for fear of the pain involved in its imagining, or of being overwhelmed by the trauma of recognition. That is why adultery, betrayal, hubris, self-performance, lies, deception, trickery, fictionality, and the propensity of the self to flee from those inner voices that whisper truths we cannot bear to hear, have been its major themes from the 18th century to the present.

In foregrounding ways in which the self is both interior and exterior, a singularity and a plurality of voices, novels might be seen to educate readers in awareness of their own confabulatory performances and, in particular, of the fallacious assumption that the ‘I’ alone has access to knowledge of itself, that we necessarily know ourselves better than we can know other people, simply because we can ‘look in’ to our own minds and not those of others. In assuming such transparency we mostly fall into error. In novels, mind is distributed—not only through the plurivocality of inner speech that renders the mind axiomatically social—but discovered to be so through the development of techniques such as free indirect discourse that hugely facilitated both double-voicing or the recognition of the affinity between the novel as a fiction and the basic fiction-making activities of the human brain as it works to fill in gaps in knowledge with its own confabulations. In George Eliot’s *Middlemarch*, for example, Dorothea Brooke’s confabulatory attempts to disguise from herself the actual mean-spirited and pedantic nature of her husband, Casaubon, and the mausoleum that is her marriage, is conveyed in that double voicing of authorial irony that we hear through her thoughts, from her first elaboration of her feelings for him: how his mind is ‘an embalmment of knowledge’, his scholarly pursuits are ‘an inscription in a door of a museum’. It is only when Dorothea can no longer avoid her feelings, which she refuses to feel, but which colour the entire feeling-tone of her *Umwelt*, her experienced world, after the trip to Rome and the return to the marriage home with its darkening interiors and atmosphere of creeping claustrophobia, that she at last recognises and can face her dreadful mistake. She comes to her senses, in finally opening up to ‘feeling, an idea wrought back to the direction of sense, like the solidity of objects’. What could be a more powerful extension of the Bayesian than that?^76^

The mind’s propensity for manufacturing pragmatic fictions and confabulatory hypotheses in the absence of certain evidence is also its capacity for inventive and abductive thinking, conjuring the possible when the probable seems unavailable through lack of evidence. The thematic preoccupation of the novel with its own condition of fictionality is therefore hardly surprising. The novel became established as a genre and disseminated itself with incredible speed from the middle of the 18th century once it discovered and explored the nature of *fictionality*: the novel may even be deemed to have invented the concept of fictionality, recognising, in an age of systems, the pragmatic truth that fictions are provisional tools for finding things out rather than mimetic representations of what they purport to describe.^77^ As Catherine Gallagher has recently argued, ‘from the outset, novelistic fictionality has been unique and paradoxical. The novel is not just one kind of fictional narrative among others; it is the kind in which and through which fictionality became manifest, explicit, widely understood, and accepted. The historical connection between the terms *novel* and *fiction* is intimate; they were mutually constitutive.’^78^ Yet even as it flaunts its fictionality, the novel also hides it by ‘locking it inside the confines of the credible’ for, if the novel is really to function as a means to expose the limitations of models and systems and to reveal how the human mind is lured into taking them for real and thereby making the world safe, closed down, convenient, the novel has to show how such models function to persuade us through their air and operational coding of probability. The moral anxiety about ‘feigning’ reality disappeared in the mid 18th century, but the fascination with laying bare the formal and conventional scaffolding of the storyworld has persisted; it seems to have most flourished during historical periods of intensified doubt and uncertainty, such as our own, when the available schema for modelling the world seem increasingly inadequate. Novels carry the Gödelian insight that all systems are incomplete, for no system can offer a complete account of itself within its own terms. The aporetic uncertainty of any system lies in its inability to get outside in order to describe itself, so that novels may have valuable things to tell us about the limits of models as systems for knowing in their careful observation of the vehicular terms of the model, their own languages and technical conventions.

One of the most compelling accounts of the novel in these terms has been put forward, perhaps not surprisingly, by a novelist. Orhan Pamuk’s *The Naive and the Sentimental Novelist* (2010), borrows from Friedrich Schiller’s conceptualisation of two fundamental modes of the poetic that Pamuk sees at the heart of the novel: the naive, which involves a transparent view of language as expressive, mimetic or referential, that seems to bring into view a pre-existing world; and what he calls the ‘sentimental’, where language points to itself as representation and therefore artifice, rendering problematic its relation to whatever is taken to be the real outside of the medium of language. Again what is strikingly apparent in this account is that the fundamentally recursive nature of the sentimental, so defined, reinforces the sense that recursivity, viewed by evolutionary anthropologists as the definitive skill of the human mind, is presented here not so much as the activity of ‘time-travelling’ and metarepresentation, as discussed earlier, but as the fundamental property without which there could be no human language at all. The branching and recursive structure of language, its implicit self-reference, is echoed in fiction’s fascination with paradoxes of self-referentiality as metareference—its preoccupation with pointing to its own impossible existence as simultaneously word and world. For the philosophical logician, the paradox of linguistic self-reference is a bothersome obstacle in the search for watertight logical categories, free of interference from the sign systems that mediate them. The logician’s favourite example is the self-referential assertion that undoes the clear separation of categories, the sentence that points to itself, such as: ‘This is not a sentence’ or the Liar Paradox, ‘All Cretans are Liars, said the Cretan’. In order to disambiguate such sentences one has to move beyond the sentence itself to a metalevel that posits a context or speaker or a further source-monitoring tag that offers resolution by presenting the sentence as existing in a novel, for example.

The paradox has fascinated rather than bothered novelists, and for almost opposite reasons, pointing to the way that language in fiction reveals a world that is entirely modelled in language and, in that sense, points only to itself and yet can seem more compelling and ‘true’ than any fully propositional sentence that is a description of the world. Schlegel’s understanding of the paradox gave rise to his concept of Romantic Irony but only after his reading of Cervantes’ *Don Quixote*, where the delusory exploits of the would-be knight are further complicated by Cervantes’ exuberant play with recursivity in the second volume. Here he presents Quixote reading the complete narrative account of his own exploits in a cheap printed book that appears impossibly adjacent in time to the events that we have just read in the story. In Pamuk’s view, the novel transports us into a storyworld, but disciplines our immersion in requiring that we reflect on the way in which we think through and therefore pay attention to the medium. This is the basis for his key argument that the novel is the definitive anti-Cartesian genre, for ‘the art of the novel relies on our ability to believe simultaneously in contradictory states … and cultivating the habit of reading novels, indicates a desire to escape the logic of the single-centred Cartesian world where mind and body, logic and imagination are placed in opposition’. The more effective the novelist is in holding in tension the naive, conceived as the empathetic and child-like, with the sentimental, conceived as the technical and self-referential, the greater and more affecting the novel: ‘the art of the novel is being able to speak about ourselves as if we were another person, and about others as if we were in their shoes. And just as there is a limit to the extent we can speak about our self as if we were another person, there is also a limit to how much we can identify with another person.’^79^

The novel’s great theme and discovery is that our commonest delusion is to believe that we have a privileged insight into our own minds; to read novels is to discover that we use the same inferential skills in reading ourselves, setting up one voice against another in our inner speech, as when we try to work out what others are thinking, desiring and believing. What seems most transparent may often emerge as most opaque. We listen in to our own inner speech but that is already constituted out of our mentalising of other minds. If we accept the anthropological argument that the mind evolved first to be social, to read other minds for threat, possibility, cooperative or competitive strategy, then it seems likely, even from this very long perspective, that the mind reads itself as a kind of ‘other’ and is no more transparent to itself, and may even be more opaque to itself, than the minds of others.^80^ In the novel, we read ourselves through the rendition and completion of the minds of others; we learn to read our own minds, therefore, against the grain of the solipsistic. That is a kind of therapy. Moreover, as a complex model that models the limits of its own complexity, the novel mostly proceeds not simply from unknowing to knowing (the usual reading of *Bildung*), but by the disconfirmation of untested and assumed hypotheses that reveal the world as ever extended and complicated by the models we use to model it; a world—as in Beck’s presentation of risk—that will ever exceed our systems of knowing. This knowledge, that renders the world both less and more knowable than we might have assumed, challenges the cognitive bias involved in trying to make the world conform to our models of it. That means trying to read the world against the grain of the egotistical and the narcissistic. That is also therapeutic.

The major theme of Wittgenstein’s later writing was the call for philosophy to provide a therapy for the deleterious effects created in adopting a broadly Cartesian outlook that foregrounds self-transparency as the key to and goal of knowledge. The ramifications of this assumption in our lives create what Wittgenstein refers to as ‘the mental cramps’ that make us sick.^81^ The problem, as he sees it, is that the very medium of language that we use to represent thought leads us into error, but such error is so deeply entangled in thinking at the level of metaphor and structural analogy, that we fail to notice its effects. Our language is awash with metaphors of visual perception as the vehicle for thinking about knowing and self-knowledge; until we begin to challenge and change the metaphors, even as we reject Cartesianism, we shall find ourselves still in its grip as the various entailments of the metaphor continue to preserve inferential relations. In other words, Wittgenstein shows how non-intentional analogical reasoning may hold us in its grip and shape and pre-structure beliefs without our realisation.^82^ So for Wittgenstein, therapy needs to happen at the level of language, through a process that leads us to become aware of the medium if we are to be cured of our erroneous expectations and the damage they create, but also to acknowledge the power of the medium to set up a world. We need a cure for the metaphors of self-transparency, the idea that we must look within and know ourselves before we can reliably look out; for mostly unknowingly, we use language to make what is abstract and conceptual and ultimately unknowable, into something that seems concrete and self-evidently before our ‘eyes’: ‘I can see it now’, ‘yes, I see’, ‘I’m in the dark’, ‘it’s getting clearer to me’; we have our eyes opened, or see something in the right light; at other times, often when we are trying to remember, we think of our minds as filing cabinets or cupboards where we ‘retrieve’ our memories as though we are looking in a cupboard and rummaging for old clothes.

Our thoughts become things that we see or touch, bats hanging in the dark as Woolf expressed it. For with her interest in and foregrounding of voice, Woolf often portrays thinking humorously, thoughts looming out of primeval darkness, like bats hanging in a cave or tumbling around like ‘old clothes in my dirty clothes basket’. In an essay entitled ‘Pictures’, she fantastically imagines the ‘mind’s eye’ turned inward like an amoebic life within, a great ‘nerve which hears and smells, which transmits heat and cold, which is attached to the brain and rouses the mind’.^83^ The grotesque concreteness of Woolf’s metaphors for thinking point to their own *reductio* effect. Woolf is puncturing her own and our yearning for transparency: for she recognises the power of the metaphor in sustaining a belief in self-transparency, the idea that we can peer into the mind like a potholer with a torch; she knew, though, that her own mind remained a mystery to her except in its *externalisation* and revelation through the voices of fiction, her fictional shaping and befriending of the voices in her head. Woolf and Wittgenstein were barely acquainted, but they might have learned valuable things from each other. Wittgenstein sought a new therapy from within philosophy to correct several hundred years of error; he desired a philosophical ‘therapy’ for all those therapies that advocate the idea of looking in and finding oneself as the beginnings of the therapeutic. Wittgenstein read few novels, or he might have found what he was searching for in their orientation to voice, decentring, complexity, fictionality and recursivity, but most of all in their awareness of language, of a medium, in shaping and building worlds. For me, that is the most overlooked yet in some ways most important contribution that the novel offers to and as therapy: a therapy for all those times and places where therapy has failed to monitor the effects of its own medium, models and metaphors.^84^

## Footnotes

1 Deleuze (1969), 237.

2 Mitchell (2013), 200.

3 Arendt (1978), 110–11.

4 Kermode (1967).

5 Connor (1997), 207.

6 See Sartre’s (1940) interesting discussion of the imagination’s ‘picturing’ power and Scarry’s (2001) use of the neuroscience of visual imagining which develops this argument.

7 Faulkner (1990), 3.

8 Woolf (1998), 3.

9 University of Liverpool (2014), a summary of the ethos of ‘shared reading’ and an explanation of the therapeutic benefits of shared reading aloud: liveness, creative inarticulacy and emergence, emotion, attention and personal and group relations. The report also contains a useful history of the project.

10 Defoe (1975), 107.

11 Ibid., 112.

12 Joyce (1960), 138.

13 Defoe, 78.

14 Ibid., 51.

15 15 Narrative therapy is the term given to a psychotherapeutic method developed by White & Epston (1990). Narrative therapy works with the broadly social constructionist idea that we constitute our identities through the available stories and narratives in our culture, and that individual problems can be reworked by externalising them through a retelling in conversation with a therapist that aims to reconfigure the main storylines.

16 See Benstock (2001).

17 Bruner (1990).

18 On reading and being transported, see Gerrig (1998) and Ryan (2001), and for a defence of what Virginia Woolf originally called the ‘common reader’ and of reading for wonder, recognition and pleasure, see Felski (2008).

19 Beck (2009), 4.

20 ‘I am not writing a system, but a history’ insists the narrator of *Tom Jones* (see Fielding ([1749] 2005), 568). For a discussion of the rise of the novel and 18th-century ‘system’ theorising see Siskin (2001).

21 Lukacs (1971).

22 See Derman (2011).

23 Bell (2000).

24 Complexity theory grew out of work developed at the Santa Fe Institute on complex adaptive systems and drew on earlier systems theorists such as Niklas Luhmann and the cybernetic theories of the 1940s and 1950s associated with Von Neuman and Shannon and Weaver. The work of Stuart Kauffman, in particular, is key for the development of complex dynamic systems theory; see Kauffman (1993); for useful discussions, see Morin (2008); Vester (2007).

25 Hacking (2006) coined the term ‘looping kinds’ to describe the way in which labelling and naming an entity can change and determine its behaviour or responses to it of others; he summarises his use of the term succinctly in a short piece.

26 See Herman (2013), Palmer (2008), Zunshine (2006) and Zunshine (2012).

27 Though one might also argue that Cervantes (2005) is intentionally equivocal too in his treatment of his protagonist’s madness, suggesting that though his nostalgia for and romantic idealisation of the code of chivalry leads him into delusional behaviours, and makes him mad, it also keeps alive a set of higher values that might otherwise die.

28 Ibid., 5.

29 Ibid.

30 Rieff (1966).

31 MacIntyre (1981), 30, argues that ‘the manager represents in his character the obliteration of the distinction between manipulative and non-manipulative social relations; the therapist represents the same obliteration in the sphere of personal life. The manager treats ends as given, as outside his scope, his concern is with technique, with effectiveness in transforming raw materials into final products … The therapist also treats ends as given, as outside his scope; his concern is with technique, with effectiveness in transforming neurotic symptoms into directed energy, maladjusted individuals into well-adjusted ones.’

32 See Jackson (2013) for a discussion of the rise and circulation of the term ‘stress’. The idea of the contemporary world as ‘unmappable’ was first put forward in Jameson’s seminal essay (1984), ‘Postmodernism and the Cultural Logic of Late Capitalism’, see Jameson (1991), 51.

33 See introduction to Weatherhead & Flaherty-Jones (2011).

34 Furedi (2004), 114.

35 Ibid., 127.

36 Ward (2012), 644; Ishiguro (1995).

37 Ishiguro, Vorda & Herzinger (1993), 31.

38 Lodge (1995), 13.

39 Franzen (2002), 73.

40 Franzen (2010), interview with Helena de Bertodano.

41 For a thorough discussion of early concerns about the fictionality of fiction, see Paige (2011).

42 Sterne (2008), 61.

43 Quoted in Toulmin (1979), 7.

44 Riley (2004), 58.

45 Vygotsky (1934) challenged Piaget’s idea of the evolution of speech as developing from egocentric to externally oriented talk. Instead, he put forward the view, based on his own experiments, that contrary to Piaget’s view, inner speech was an internalisation of voiced egocentric speech, acquired socially and internalised and condensed as silent verbal thought, suggesting that thinking as inner speech is an important factor in constructing a self out of the conversations and verbal relations conducted in the social world. The external world is thus always already a constituent of the internal verbal world of thought. On Vygotsky’s theory of inner speech and its relevance to understanding the phenomenon of auditory verbal hallucinations, see Fernyhough & McCarthy Jones (2010), 87–105.

46 Bakhtin (1981), 219.

47 Kuzmicova (2013).

48 Toulmin (1979), 5, 7.

49 Beckett (1967), 91–2.

50 For an excellent recent discussion of this aspect of Beckett’s work, see Salisbury (2012).

51 Wells (1922), 8.

52 Ibid., 6.

53 Ibid., 10.

54 Ibid., 6–7.

55 See Jackson (2013). Mark Jackson points out the fact that Wells’s novel is published the year of the report on shell-shock. Shephard (2014) has a very thorough discussion of the report and its impact.

56 Woolf (1925), 4, 58.

57 Ibid., 57.

58 Ibid., 93.

59 Herman (1992); Karuth (1995). Bessel van der Kolk’s work drew substantially on the much earlier work of Pierre Janet on dissociation and voices, which had largely been neglected since the early 20th century as a consequence of the prominence given to Freudian theory. Janet’s work developed the idea of 19th-century physiological psychology on the idea of an ‘intelligent unconscious’, the idea that parts of the self split off through trauma returned as voices in states of dissociation bearing crucial truths previously unacknowledged by the hearer.

60 Recent psychological research suggests a process very like Woolf’s portrait in her novel; see, for example, Bentall, de Sousa, Varese, Wickham, Sitko, Haarmans & Read (2014), 1011–22.

61 Early discussions drew heavily on 19th-century work in physiology and psychology, such as Spencer (1855), and on early 20th-century medical debates around neurasthenia and ‘nervousness’.

62 Selye (1946), 117–230; this work was influenced by that of Harvard physiologist Walter B. Cannon (1932).

63 Bell, ed. (1977–84), ii, 248.

64 See DeSalvo (1989); Trombley (1981).

65 Woolf writes frequently of racing thoughts in her diary and fictionalises the experience near the beginning of *To the Lighthouse* (1927) in the strange entanglement of Lily’s thoughts with the sudden sound of the detonation of Jasper’s gun, so her thoughts appear as if a ‘black cloud of starlings’ emanating from her brain.

66 Woolf (2002), 93.

67 Woolf (1958), 42, 23.

68 Recent developments in therapeutic interventions that draw on such insights include narrative and avatar therapy. See White & Epston (1992) for an account by the pioneers of narrative therapy; the psychiatrist Julian Leff has recently pioneered avatar therapy, see Leff, Williams, Huckvale, Arbuthnot & Leff (2014).

69 Woolf ([1927], 1960), 102–3.

70 Woolf (1986–94), 85.

71 Bakhtin (1984), 189.

72 This idea is discussed by Laing (1960).

73 Bakhtin (1984), 195.

74 This Bayesian idea of the mind has been developed in recent theories of the ‘predictive mind’; see Clark (2013), 181–204; for a summary of the work on the predictive mind to date, see Hohwy (2013); for an account of the legacy of the work of the Reverend Thomas Bayes in the 18th century, see McGrayne (1911).

75 Gass (1983), 60.

76 Eliot (2004), 193.

77 The classic work on this is Vaihinger (1911).

78 Gallagher (2006), i. 337.

79 Pamuk (2010), 33, 71.

80 See Tomasello (2014).

81 Wittgenstein (1998); on the emergence of Wittgenstein’s thinking about philosophy of therapy, see Fisher (2011) and Pateman (1992).

82 This might be seen as a factor in the perpetuation but reversal of Cartesian thinking engaged in Ian Hacking’s argument that in an age of materialist reductionism and assumptions that the mind is reducible to the brain, we are living in an era of reverse or neo-Cartesianism, where the mind is seen as driven by the body rather than vice versa: see Hacking (2007), 78–105.

83 Woolf (1986–94) iv. 244, 519.

84 The research for this essay was supported by a Wellcome Trust strategic award (WT098455MA) and a Leverhulme award (F/DO128?BF.
